# Characteristics of NH_4_^+^ and NO_3_^−^ fluxes in tea (*Camellia sinensis*) roots measured by scanning ion-selective electrode technique

**DOI:** 10.1038/srep38370

**Published:** 2016-12-05

**Authors:** Li Ruan, Kang Wei, Liyuan Wang, Hao Cheng, Fen Zhang, Liyun Wu, Peixian Bai, Chengcai Zhang

**Affiliations:** 1 National Center for Tea Improvement, Tea Research Institute, Chinese Academy of Agricultural Sciences; Key Laboratory of Tea Plant Biology and Resources Utilization, Ministry of Agriculture, Hangzhou 310008, China; 2 State Key Laboratory of Soil and Sustainable Agriculture, Institute of Soil Science, Chinese Academy of Sciences, Nanjing, 210008, China

## Abstract

As a vital beverage crop, tea has been extensively planted in tropical and subtropical regions. Nitrogen (N) levels and forms are closely related to tea quality. Based on different N levels and forms, we studied changes in NO_3_^−^ and NH_4_^+^ fluxes in tea roots utilizing scanning ion-selective electrode technique. Our results showed that under both single and mixed N forms, influx rates of NO_3_^−^ were much lower than those of NH_4_^+^, suggesting a preference for NH_4_^+^ in tea. With the increase in N concentration, the influx rate of NO_3_^−^ increased more than that of NH_4_^+^. The NH_4_^+^ influx rates in a solution without NO_3_^−^ were much higher than those in a solution with NO_3_^−^, while the NO_3_^−^ influx rates in a solution without NH_4_^+^ were much lower than those in a solution with NH_4_^+^. We concluded that (1) tea roots showed a preference for NH_4_^+^, (2) presence of NO_3_^−^ had a negative effect on NH_4_^+^ influx, and (3) NH_4_^+^ had a positive effect on NO_3_^−^ influx. Our findings not only may help advance hydroponic tea experiments but also may be used to develop efficient fertilization protocols for soil-grown tea in the future.

As a crucial component of chlorophylls, nucleic acids, proteins and a great number of secondary plant metabolites, nitrogen (N) is essential for the growth of plants. Nitrate (NO_3_^−^) and ammonium (NH_4_^+^) are two major inorganic N forms for plants in soils. Due to various factors (e.g., root interference, soil moisture, soil microorganisms, etc.), the reciprocal transformation between ammonium and nitrate is very common in soils^1^. Thus, roots are always ready to absorb both forms of nitrogen in soils. Both ions can be absorbed and used by plants because root cells possess transport systems such as nitrate and ammonium transporters^2^. NO_3_^−^ and NH_4_^+^ have different biochemical and energetic features for assimilation, leading to various net fluxes of NO_3_^−^/NH_4_^+^ and ion preferences of plants, although both ions can be used by plants^3^.

Comparative studies on net fluxes of NH_4_^+^ and NO_3_^−^ have been conducted in different plants, and the preference for NH_4_^+^ or NO_3_^−^ is usually associated with the physiological needs of plants in various ecosystems^4^. Tea is an important beverage crop that has been extensively planted in tropical and subtropical regions. In tea plants, N levels and forms, especially in young shoots, are associated with the quality of tea. Previous research has demonstrated that tea plants have a higher absorption of NH_4_^+^ compared to NO_3_^−^ 5. However, high concentrations of NH_4_^+^ are toxic in a majority of plants, including woody plants. If only NO_3_^−^ or both ions are provided, no detrimental influences can be detected in plants^6^,^7^. Little information can be found on the interactions between NH_4_^+^ and NO_3_^−^ fluxes in tea roots, although the uptake of NH_4_^+^ and NO_3_^−^ in tea has been explored extensively^4^,^5^,^8^. Moreover, most previous studies on N uptake were carried out using an ^15^N labeling method, which was unable to interpret the dynamic processes of NH_4_^+^ and NO_3_^−^ fluxes^9^,^10^.

Taking an electrophysiological approach, scanning ion-selective electrode technique (SIET) can evaluate ion/molecule-specific activities non-invasively^11^. To date, NH_4_^+^, NO_3_^−^, H^+^, Cd^2+^, Ca^2+^, Mg^2+^, Na^+^, Cl^−^, K^+^, O_2_ and Al^3+^ have been identified utilizing SIET; however, the application of SIET for the examinations of net NH_4_^+^ and NO_3_^−^ fluxes in tea roots has not yet been reported.

In this study, the fluxes of net NH_4_^+^ and NO_3_^−^ in absorbing tea roots exposed to various N forms were evaluated with SIET non-invasively. This research had the following objectivities: (1) to monitor any alterations in net NH_4_^+^ and NO_3_^−^ fluxes in tea roots under different N forms, and (2) to assess the interaction between fluxes of NH_4_^+^ and NO_3_^−^ in tea roots. This research is the first attempt to identify fluxes of net NH_4_^+^ and NO_3_^−^ in the presence of different N forms and interactions between NH_4_^+^ and NO_3_^−^ fluxes in tea roots utilizing SIET. Our findings may help advance hydroponic tea experiments and effective fertilization protocols for future soil-grown tea plants.

## Results

### Net fluxes of NO_3_^−^ and NH_4_^+^ under different N forms and levels

**Figure 1:**
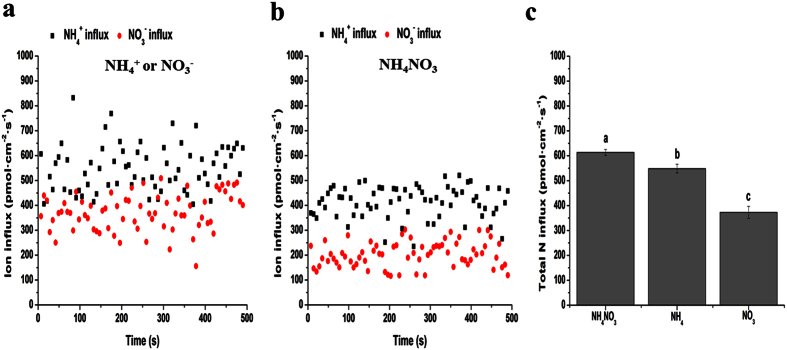
Net fluxes of NO_3_^−^ and NH_4_^+^ on surfaces of tea roots under different N forms. Net fluxes of NO_3_^−^ and NH_4_^+^ on tea root surfaces under single (**a**) and mixed N forms (**b**). Total N influx rates under the different N forms (**c**). The mean ± SE (n = 6) is shown in the data. In order to eliminate the “noise” caused by the oscillation, not only 6 biological repetitions, but also 70 measurement time points in each repetition were considered. Thus, SE = SD/√420. The different letters indicate differences between means at *P* < 0.05.

Tea roots were immersed in measuring solutions with different N forms (1 mM NH_4_NO_3_, 2 mM KNO_3_ or 1 mM (NH_4_)_2_SO_4_) to monitor the net fluxes of NO_3_^−^ and NH_4_^+^ under various N forms. Net flux curves of NH_4_^+^ and NO_3_^−^ are shown in Fig. 1. After the 7 d N starvation treatment, both NH_4_^+^ and NO_3_^−^ presented influx states on the root surface when different N forms were given. In addition, the influx rates of NO_3_^−^ and NH_4_^+^ improved gradually. In comparison to the treatments of a single N form, the influx rates of NH_4_^+^ and NO_3_^−^ under the NH_4_NO_3_ treatment were more stable (Fig. 1a,b). The influx rates of NO_3_^−^ were lower than those of NH_4_^+^ under both single and mixed N form treatments, which suggested that tea roots had a preference for NH_4_^+^ (Fig. 1a,b). When N levels were the same, the total N influx rate of the NH_4_NO_3_ treatment was considerably higher than that of the single N form treatments (Fig. 1c).

**Figure 2:**
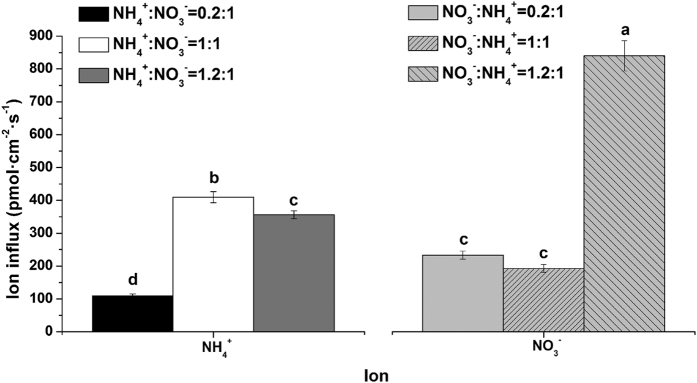
Influx rates of NH_4_^+^ and NO_3_^−^ on tea root surfaces under different proportion of N sources. The mean ± SE (n = 6) is shown in the data. The different letters indicate differences between means at *P* < 0.05.

The influx rates of NO_3_^−^ and NH_4_^+^ under various proportions of N sources are shown in Fig. 2. With an increase in the NH_4_^+^ concentration, the influx rates of NH_4_^+^ first increased and then decreased under the same concentration of NO_3_^−^ (Fig. 2). The highest influx rate of NH_4_^+^ appeared when the ratio of NH_4_^+^:NO_3_^−^ was 1:1. For the influx rates of NO_3_^−^, the highest influx rate of NO_3_^−^ appeared when the ratio of NO_3_^−^:NH_4_^+^ was 1.2:1. This suggested that NH_4_^+^:NO_3_^−^ at 1:1 was the critical point and that the absorption rate of NH_4_^+^ might not improve with an increase of NH_4_^+^; meanwhile, the variation of the NO_3_^−^ influx rate was extremely different.

**Figure 3:**
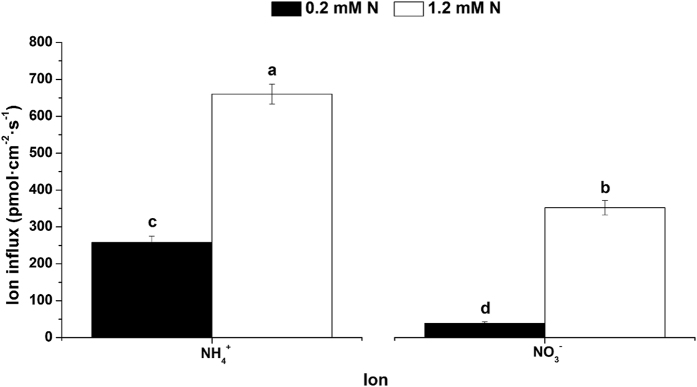
Influx rates of NH_4_^+^ and NO_3_^−^ on tea root surfaces under different N levels. The mean ± SE (n = 6) is shown in the data. The different letters indicate differences between means at *P* < 0.05.

The influx rates of NH_4_^+^ and NO_3_^−^ under different N levels are shown in Fig. 3. The influx rates of NH_4_^+^ were 6.69 and 1.87 times higher compared to NO_3_^−^ at 0.2 and 1.2 mM N levels, respectively. With increasing N concentration, the influx rates of NO_3_^−^ and NH_4_^+^ improved significantly. In addition, the influx rate of NO_3_^−^ improved more than NH_4_^+^ with the increase in N concentration. Although a high concentration of ammonium N is toxic for a majority of plants (including woody plants), the high concentration of NH_4_^+^ (1.2 mM) did not affect tea tree growth in this study (Supplementary Figures S1 and 2). From phenotyping data, biomass and N contents of root, stem and leaf were higher with the supply of NH_4_^+^-N compared to the supply of NO_3_^−^-N. Thus, tea trees had better growth with the supply of NH_4_^+^-N compared to the supply of NO_3_^−^-N (Supplementary Figure S1). In addition, the content of total free amino acid in the supply of NH_4_^+^-N was higher compared to the supply of NO_3_^−^-N (Supplementary Figure S2). The main amino acids of the tea tree (such as aspartic acid, glutamic acid and theanine) were higher with the supply of NH_4_^+^-N compared to the supply of NO_3_^−^-N (Supplementary Figure S2). This suggested that tea roots had a stronger NH_4_^+^ uptake ability, especially under low N conditions.

### Interactions between NH_4_^+^ and NO_3_^−^ fluxes in tea roots

**Figure 4:**
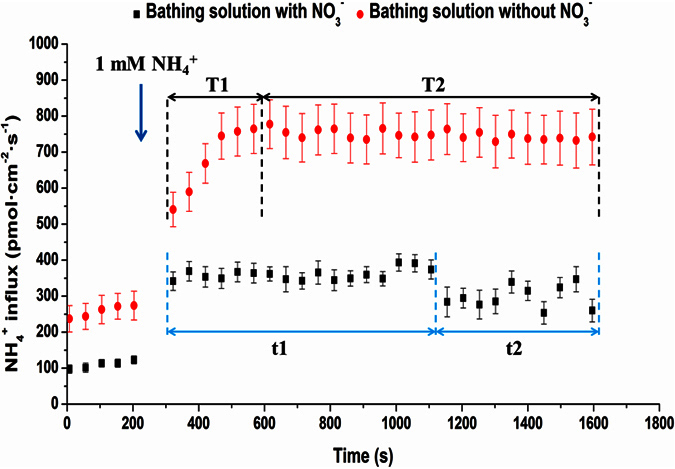
Influence of NO_3_^−^ on NH_4_^+^ net fluxes on tea root surfaces. After adding (NH_4_)_2_SO_4_ to the bathing solution without or with 1 mM KNO_3_, the changes in tea root NH_4_^+^ net fluxes (averaged over 49 s) are presented. The mean ± SE of NH_4_^+^ influxes during the measurement period are shown (n = 6). (NH_4_)_2_SO_4_ was added at the vertical arrows.

Changes in NH_4_^+^ flux are shown in Fig. 4 after adding NH_4_^+^ to the bathing solution either with or without NO_3_^−^. NH_4_^+^ presented influx states on the root surface regardless of whether the bathing solution had NO_3_^−^. The NH_4_^+^ influx rates in the bathing solution without NO_3_^−^ were much higher compared with those in a solution with NO_3_^−^. In plants without NO_3_^−^ supply, the influx rates of NH_4_^+^ increased and peaked approximately 200 s after NH_4_^+^ addition (T1 stage), suggesting a vibrant status of the influx system without the NO_3_^–^ supply and the ability to retain cytoplasmic NH_4_^+^ to a specific degree in tea roots. The influx rates of NH_4_^+^ remained stable (T2 stage), demonstrating that the NH_4_^+^ influx and efflux systems had reached a balance and that NH_4_^+^ influx was dominant. In plants with the NO_3_^−^ supply, the influx rates of NH_4_^+^ did not increase; however, they stabilized quickly and maintained the rate (t1 stage). Approximately 800 s after NH_4_^+^ addition, the influx rates of NH_4_^+^ began to decrease (t2 stage). The NH_4_^+^ influx rates in the bathing solution with or without K^+^ are shown in Supplementary Figure S3. There was little difference between the NH_4_^+^ influx rates in the bathing solution with or without K^+^, indicating that adding K^+^ had little effect on the net flux of NH_4_^+^ in this study.

**Figure 5:**
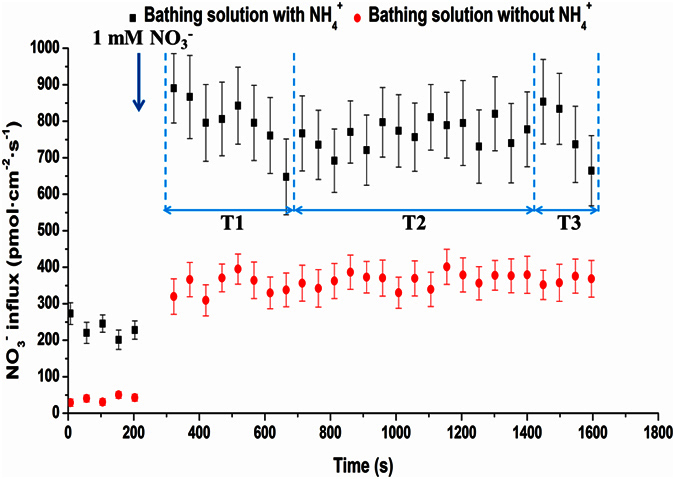
Influence of NH_4_^+^ on NO_3_^−^ net fluxes on tea root surfaces. Variations of NO_3_^−^ net fluxes in tea roots (averaged over 49 s) after adding KNO_3_ to the bathing solution without or with 0.5 mM (NH_4_)_2_SO_4_ are presented. The mean ± SE of NO_3_^−^ influxes during the measurement period are shown (n = 6). KNO_3_ was added at the vertical arrows.

Changes in NO_3_^−^ flux are shown in Fig. 5 after adding NO_3_^−^ to the bathing solution without or with NH_4_^+^. NO_3_^−^ presented influx states on the root surface regardless of whether the bathing solution had NH_4_^+^. The NO_3_^−^ influx rates in the bathing solution without NH_4_^+^ were much lower compared to those in a solution with NO_3_^−^, which were just the opposite of the NH_4_^+^ influxes above. In plants without the NH_4_^+^ supply, the influx rates of NO_3_^−^ remained stable. However, with the NH_4_^+^ supply, the influx rates of NO_3_^−^ could be divided into three stages: (1) a decrease with the lowest point approximately 400 s after NH_4_^+^ addition (T1 stage); (2) a spiral increase from approximately 400 to 1200 s after NH_4_^+^ addition (T2 stage); and (3) a slope decrease (T3 stage). The influx rates of NO_3_^−^ were more unstable compared to NH_4_^+^, especially in the bathing solution with NH_4_^+^.

## Discussion

In all treatments, tea roots showed absorption states of NH_4_^+^ and NO_3_^−^, showing that the thresholds for plant development were higher than the cytosolic concentrations of NH_4_^+^ and NO_3_ after a 7 d N deprivation. Tea roots needed to maintain a certain level of NH_4_^+^ or NO_3_^−^ in the cytoplasm^7^. Additionally, tea roots showed a preference for NH_4_^+^ when NH_4_^+^ and NO_3_^−^ existed at the same time. Greater net uptake of NH_4_^+^ compared to net uptake of NO_3_^−^ were reported in maize, rice and wheat roots when NH_4_^+^ and NO_3_^−^ were supplied simultaneously^12^,^13^,^14^. In comparison to NO_3_^−^ influx, there were some possible reasons for the observed preference for NH_4_^+^ influx. As various root tissues needed various amounts of NH_4_^+^ and NO_3_^−^, one reason might involve root morphology. A higher concentration of NH_4_^+^ was needed for protein synthesis in the meristem zone^12^. In addition, NH_4_^+^ absorbed by plants was transformed to amino acids directly in the roots, which required less energy and reducing equivalents for assimilation and transportation in most species^15^,^16^. For tea plants, ammonia supplied to the tea roots was quickly stored as theanine, glutamine and arginine in the roots and leaves before the sprouting new shoots^17^. Previous research^18^ has reported that after tea plants were fed with ^15^N-NO_3_^−^ and ^15^N-NH_4_^+^, the amount of total amino acid in the xylem sap significantly increased, and those fed with ^15^N-NH_4_^+^ had a greater increase compared to those fed ^15^N-NO_3_^−^. Different from other plants, tea can turn redundant glutamic acid into theanine, which was a peculiar amino acid in tea^19^. Moreover, NH_4_^+^ was more readily assimilated than NO_3_^−^ into theanine^20^. This process might have eliminated NH_4_^+^ toxicity in tea roots and have created a NH_4_^+^ preference in tea^5^.

Previous studies demonstrated that crop growth and yield were significantly improved when two forms of nitrogen were supplied at the same time. In this study, the highest total N influx rates were observed with the NH_4_NO_3_ treatment when N levels were the same, which suggested that tea roots had the highest nitrogen absorption efficiency when two forms of nitrogen were supplied simultaneously. This result was consistent with other plants reported^14^,^21^,^22^. For most plants, roots released H^+^ after absorption of NH_4_^+^, leading to a decreased pH in the growth medium, while roots released OH^−^ after absorption of NO_3_^−^, leading to the increased pH in the growth medium^23^,^24^,^25^. A mixed application of NO_3_^−^ and NH_4_^+^ at a 1:1 ratio encouraged higher foliar N content and glutamine synthetase (GS) and glutamate synthase (GOGAT) activity in tea^26^. Although tea roots preferred NH_4_^+^, a single application of ammonium nitrogen could aggravate soil acidification in tea gardens^27^, and could significantly raise the amount of aluminum taken up by tea plants, leading to a decrease in tea quality^28^. Therefore, to get a higher N absorption efficiency in tea and reduce soil acidification in tea gardens, two forms of nitrogen should be supplied simultaneously.

With the increase in N concentration, the influx rate of NO_3_^−^ was improved more than NH_4_^+^, which might be contributed to differences in activities and expressions of the transport systems between the two ions. Net NO_3_^−^ and NH_4_^+^ absorption can be regulated by low-affinity (LATS) and high-affinity transporters (HATS). When the exterior NH_4_^+^ concentration was below 1 mM, HATS played a leading role in the uptake of NH_4_^+^ absorption, and when the exterior NH_4_^+^ concentration was above 1 mM, LATS were activated^29^. While HATS played a main role in regulating NO_3_^−^ uptake when the external NO_3_^−^ concentration was below 1 mM, LATS started to work when the external NO_3_^−^ concentration was above 0.5 mM^30^. According to previous studies, with the increase in N concentration, the LATS for NO_3_^−^ were stimulated much earlier than the LATS for NH_4_^+^. In addition, Glass *et al*.^31^ and Britto *et al*.^32^ reported that transport through the low-affinity systems were poorly regulated when the high-affinity NH_4_^+^ fluxes were effectively regulated. This might lead to the massive vain cycling of NH_4_^+^ across the plasma membrane and toxic effects of superfluous NH_4_^+^ accumulation. Thus, the influx rate of NO_3_^−^ was improved more than NH_4_^+^ with the increase in N concentration. The present data showed that the influx rate of NO_3_^−^ was significantly lower than the influx rate of NH_4_+ under low N conditions (0.2 mM N), which might be contributed to a lower energy cost for both transport and assimilation of NH_4_^+^ 16.

**Figure 6:**
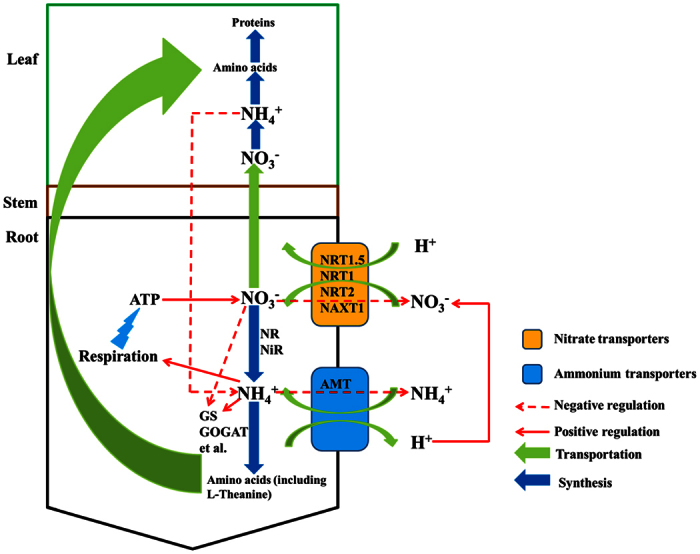
Proposed mechanisms of interaction between NH_4_^+^ and NO_3_^−^ fluxes in tea roots. The absorption and transformation processes of NH_4_^+^ and NO_3_^−^ are shown. Some influence factors of NH_4_^+^ and NO_3_^−^ absorption are listed to explain possible mechanisms of interaction between the NH_4_^+^ and NO_3_^−^ fluxes in tea roots.

The present data demonstrates that the presence of NO_3_^−^ had a negative effect on net NH_4_^+^ uptake. Before being assimilated by plants, NO_3_^−^ was restored as NH_4_^+^, leading to the increase NH_4_^+^ concentration in the cytoplasm. The HATS of NH_4_^+^ were influenced by the negative feedback regulations and an increased cytosolic NH_4_^+^ concentration suppressed the root influx of NH_4_^+^ 31. As widely acknowledged, NO_3_^−^ is a mobile ion and can be restored both in the roots and leaf. Nitrate in tea roots can be directly transported to the xylem sap and then to the leaf^18^. When NO_3_^−^ was restored as NH_4_^+^ in the leaf, the concentration of NH_4_^+^ in the leaf would increase. A shoot-to-root signal might be regarded as the effect of the local N status that controls the influx of NH_4_^+^ 33. In addition, NO_3_^−^ had an inhibitory influence on GS enzyme activity, which might also a reason for the negative effect of NO_3_^−^ on NH_4_^+^ influx^34^,^35^ (Fig. 6).

In contrast, the presence of NH_4_^+^ had a positive effect on net NO_3_^−^ uptake, which was consistent with previous studies performed in other species^14^,^33^,^36^. There were several reasons to support this result. First, NH_4_^+^ has been reported to increase the respiration rate of plants, which can provide energy for NO_3_^−^ uptake^37^. Second, the balance of H^+^, NH_4_^+^ and NO_3_^−^ could be used to explain this result. Tea roots took up a large amount of NH_4_^+^ during growth and later released H^+^ to maintain the charge balance in the plant body^38^. According to our current understanding of NO_3_^−^ transportation, NO_3_^−^ influx occurs with one H^+^ symport, and two possible H^+^ ions promote the inward transportation of one NO_3_^−^ ion, while the efflux of H^+^ is meant to balance the influx of NH_4_^+^. According to previous studies, due to NH_4_^+^ stimulation of H^+^ efflux, a stimulation of NO_3_^−^ absorption by NH_4_^+^ might increase the availability of H^+^ for co-transport^39^,^40^. Moreover, NH_4_^+^ could significantly increase the activities of the GS and GOGAT enzymes, which plays an important role in nitrate reduction and nitrogen assimilation, providing material bases for NO_3_^−^ absorption^34^. In our study, the NO_3_^−^ influx rates were more irregular in the various treatments. This was because NO_3_^−^ could develop the functions of a mobile ion and an osmoticum^14^ (Fig. 6).

In conclusion, the elucidation of the mechanisms related to N transport is difficult when assessing net N flux. Net N flux is established as the total of N efflux and influx. Additionally, net N flux is affected by transportation and assimilation rates. The findings showed that tea roots presented influx states of NH_4_^+^ and NO_3_^−^ after a 7 d N-starvation. The uptake rates of NH_4_^+^ in tea plants were higher than those of NO_3_^−^. NH_4_^+^-N can make tea trees grow better when only one single N source can be provided. Furthermore, the presence of NO_3_^−^ had a negative effect on net NH_4_^+^ influx, while NH_4_^+^ had a positive influence on net NO_3_^−^ influx. These findings may not only help guide further hydroponic experiments with tea but also help in developing efficient fertilization protocols for field-grown tea.

## Methods

### Plant materials and cultivation

*Camellia sinensis* var. Longjing 43 was used in this study. Annual cutting seedlings of Longjing 43 were transplanted to a full-strength nutrient solution for 75 d. The full-strength nutrient solution contained macronutrients (mmol L^−1^) NH_4_NO_3_ (1), KH_2_PO_4_ (0.07), K_2_SO_4_ (0.3), MgSO_4_·7H_2_O (0.67), CaCl_2_·2H_2_O (0.53), and Al_2_(SO_4_)_3_·18H_2_O (0.035) and micronutrients (μmol L^−1^) H_3_BO_4_ (7), MnSO_4_·H_2_O (1), ZnSO_4_·7H_2_O (0.67), CuSO_4_·5H_2_O (0.13), (NH_4_)_6_Mo_7_O_24_·4H_2_O (0.047) and EDTA-Fe (4.2) at pH 5.0. The nutrient solution was circulated by pumps for 24 h every day and replaced every 3 days. Next, an N starvation treatment was carried out for 7 d. The N starvation treatment was conducted using the following nutrient solution which contained macronutrients (mmol L^−1^) KH_2_PO_4_ (0.07), K_2_SO_4_ (0.3), MgSO_4_·7H_2_O (0.67), CaCl_2_·2H_2_O (0.53), and Al_2_(SO_4_)_3_·18H_2_O (0.035) and micronutrients (μmol L^−1^) H_3_BO_4_ (7), MnSO_4_·H_2_O (1), ZnSO_4_·7H_2_O (0.67), CuSO_4_·5H_2_O (0.13), (NH_4_)_6_Mo_7_O_24_·4H_2_O (0.047) and EDTA-Fe (4.2) at pH 5.0^20^. The nutrient solution was circulated by pumps for 24 h every day and replaced every three days. After the 7 d N starvation treatment, the seedlings were harvested to measure ion fluxes.

### Determinations of NO_3_^−^ and NH_4_^+^ fluxes at the root surface

The absorbing tea roots were chosen and cut off from the root system of every plant in every treatment group to evaluate the net fluxes of NO_3_^−^ and NH_4_^+^ in tea roots under various N forms. For the different nitrogen form treatments, tea roots were immersed in measuring solutions with different N forms (NH_4_NO_3_^−^N: 0.1 mM CaSO_4_, 1 mM NH_4_NO_3_ and 0.3 mM MES; NH_4_^+^-N: 0.1 mM CaSO_4_, 1 mM (NH_4_)_2_SO_4_, and 0.3 mM MES; and NO_3_^−^-N: 0.1 mM CaSO_4_, 2 mM KNO_3_ and 0.3 mM MES). MES is 2-(N-morpholino)ethanesulfonic acid hydrate buffer. For different N level treatments, tea roots were soaked in measuring solutions with different N levels (0.2 mM NH_4_^+^-N: 0.3 mM MES, 0.1 mM CaSO_4_ and 0.1 mM (NH_4_)_2_SO_4_; 1.2 mM NH_4_^+^-N: 0.3 mM MES, 0.1 mM CaSO_4_ and 0.6 mM (NH_4_)_2_SO_4_; 0.2 mM NO_3_^−^-N: 0.3 mM MES, 0.1 mM CaSO_4_ and 0.2 mM KNO_3_; and 1.2 mM NO_3_^−^-N: 0.3 mM MES, 0.1 mM CaSO_4_ and 1.2 mM KNO_3_). Before analysis, tea roots were transferred to Petri dishes containing 10 mL of measuring solution and equilibrated for 10 min to reduce possible transition effects due to changes in the environmental conditions. Next, the equilibrated root was moved to another Petri dish containing fresh measuring solution and either NH_4_^+^ or NO_3_^−^ flux was measured utilizing the SIET technique. The coefficient of variation under different balance times were shown in Supplementary Figure S4. When the pretreatment time was 10 min, the coefficient of variations under the different treatments was lower and more stable. Therefore, a 10-min pretreatment time was enough and suitable for our study. Six repetitions were established for each treatment. In order to determine the area along the root axis corresponding with maximal net NH_4_^+^ and NO_3_^−^ influx, the net fluxes of both ions were measured along the root tips to an area located 40 mm from the apex (Figure S5). The maximum net NH_4_^+^ and NO_3_^−^ influxes occurred in area between 15 and 25 mm from the root apex. Thus, we chose area between 15 and 25 mm from the root apex as the measurement site. The measuring time of each root was 10 min. The SIET technique was used to measure the net ion flux (NMT-NRP-00A00 system, Younger USA Science and Technology Corporation). The SIET system and the corresponding application process have been previously described in detail for ion flux detection^14^. Briefly, ion-selective microelectrodes designed with 2–4 μm apertures were manufactured and silanized (for the NH_4_^+^ electrode, 100 mM NH_4_Cl was used as a backfilling solution, followed by a NH_4_^+^ selective liquid ion exchange cocktail (#09879, Sigma); for the NO_3_^−^ electrode, 10 mM KNO_3_ was used as the backfilling solution, followed by a NO_3_^−^ selective liquid ion exchange cocktail (#72549, Sigma)). Prior to performing the flux measurements, the microelectrodes were calibrated^14^.

The absorbing roots of tea were soaked in a test solution and excised from the root system to evaluate the effect of NO_3_^−^ on NH_4_^+^ flux (D (with NO_3_^−^): 0.3 mM MES, 0.1 mM CaSO_4_, 0.1 mM (NH_4_)_2_SO_4_, and 1 mM KNO_3_; E (without NO_3_^−^): 0.3 mM MES, 0.1 mM CaSO_4_ and 0.1 mM (NH_4_)_2_SO_4_). NH_4_^+^ flux was measured utilizing the SIET technique for 5 min after a 10-min balance in the measuring solution. Next, 0.5 mM (NH_4_)_2_SO_4_ was added to the measuring solution. After each addition of (NH_4_)_2_SO_4_, during the first 1–2 min, the measuring solution was mixed thoroughly by expelling and sucking it into a pipette 10 times. NH_4_^+^ flux was measured using the SIET technique for another 25 min. The unstable data during the early stage were removed to gain ion flux curves.

The absorbing roots of tea were immersed in a measuring solution and excised from the root system to study the effect of NH_4_^+^ on NO_3_^−^ flux (F (with NH_4_^+^): 0.3 mM MES, 0.1 mM CaSO_4_, 0.2 mM KNO_3_, and 0.5 mM (NH_4_)_2_SO_4_; G (without NH_4_^+^): 0.3 mM MES, 0.1 mM CaSO_4_ and 0.2 mM KNO_3_). NO_3_^−^ flux was measured utilizing the SIET technique for 5 min after a 10-min balance in the measuring solution. Next, 1.0 mM KNO_3_ was added to the measuring solution. The test process was the same as above.

### Statistical analysis

To verify the importance of differences between treatments, one-way ANOVA was performed. Microsoft Excel (Microsoft Corporation, USA) and SPSS Window version 17 (SPSS Incorporation, Chicago, USA) were used to analyze data. To draw figures for the data, OriginPro 8.1 (Origin Incorporation, Chicago, USA) was utilized.

## Additional Information

**How to cite this article**: Ruan, L. *et al*. Characteristics of NH_4_^+^ and NO_3_^−^ fluxes in tea (*Camellia sinensis*) roots measured by scanning ion-selective electrode technique. *Sci. Rep.* **6**, 38370; doi: 10.1038/srep38370 (2016).

**Publisher's note:** Springer Nature remains neutral with regard to jurisdictional claims in published maps and institutional affiliations.

## Supplementary Material

Supplementary Information
