# Supplementary material for: Characteristics of NH4+ and NO3− fluxes in tea (Camellia sinensis) roots measured by scanning ion-selective electrode technique
Source: Sci Rep. 2016 Dec 5;6:38370. doi: 10.1038/srep38370 (PMC5137579; doi:10.1038/srep38370)

Characteristics of NH_4^+ and NO_3^- fluxes in tea (*Camellia sinensis*) roots measured by scanning ion-selective electrode technique

Li Ruan[1, 2], Kang Wei[1], Liyuan Wang[1*], Hao Cheng[1*], Fen Zhang[1], Liyun Wu[1], Peixian Bai[1] & Chengcai Zhang[1]

[1]National Center for Tea Improvement, Tea Research Institute, Chinese Academy of Agricultural Sciences, Hangzhou 310008, China,
[2]State Key Laboratory of Soil and Sustainable Agriculture, Institute of Soil Science, Chinese Academy of Sciences, Nanjing, 210008, China.

*Corresponding Author
Prof. Liyuan Wang and Prof. Hao Cheng
National Center for Tea Improvement
Tea Research Institute
Chinese Academy of Agricultural Sciences
Hangzhou 310008, China
E-mail: wang-liyuan@hotmail.com
Tel: +86 0571 86650575
Fax: +86 0571 86653177

Supplementary Information

Supplementary Figure S1: Phenotypes (a), plant biomass (b) and tissue N content (c) of tea seedlings under different N forms (1.2 mM NO_3^--N and 1.2 mM NH_4^+-N).

Supplementary Figure S2: The content of free amino acids in tea leaves under different N forms (1.2 mM NO_3^--N and 1.2 mM NH_4^+-N).

Supplementary Figure S3: Influence of K^+ on NH_4^+ net fluxes on tea root surfaces.

Supplementary Figure S4: The coefficient of variation under different balance time.

Supplementary Figure S5: Net NH_4^+ (a) andNO_3^- (b) fluxes along root tips of tea.

Supplementary Figure S6: Original recordings of all 6 biological replicates for NH_4^+ influx under single N form treatment.

Supplementary Figure S1: Phenotypes (a), plant biomass (b) and tissue N content (c) of tea seedlings under different N forms (1.2 mM NO_3^--N and 1.2 mM NH_4^+-N).

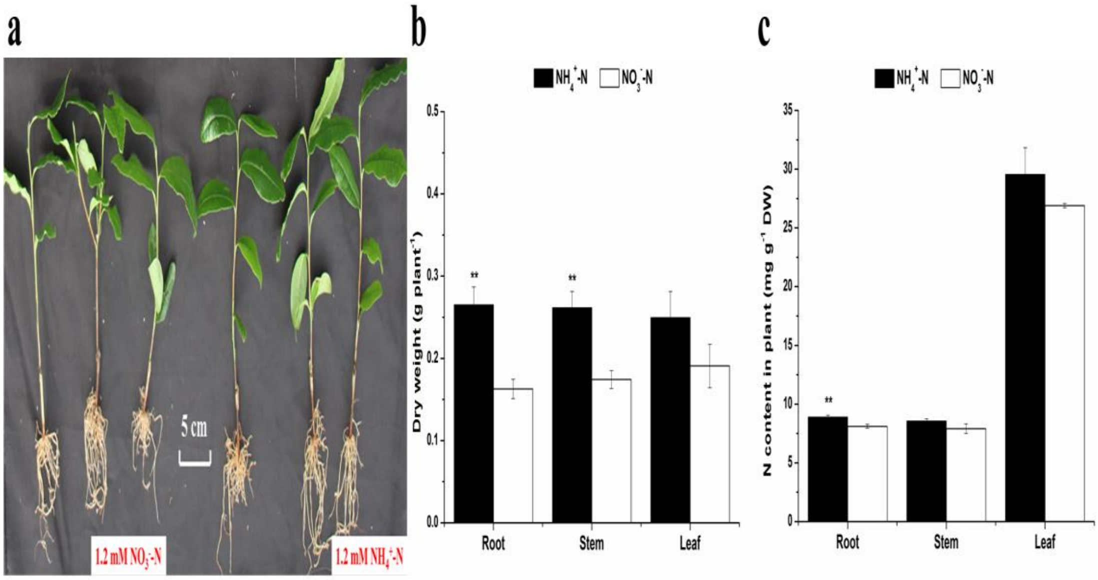

Supplementary Figure S2: The content of free amino acids in tea leaves under different N forms (1.2 mM NO_3^--N and 1.2 mM NH_4^+-N).

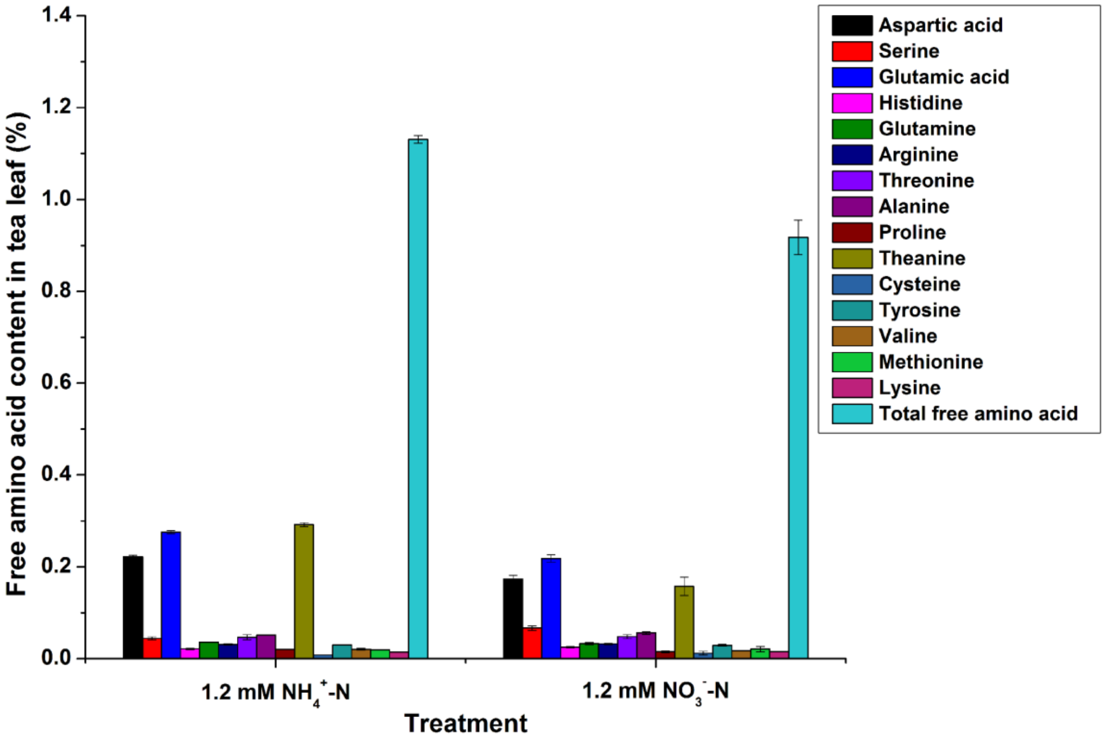

Supplementary Figure S3: Influence of K^+ on NH_4^+ net fluxes on tea root surfaces.

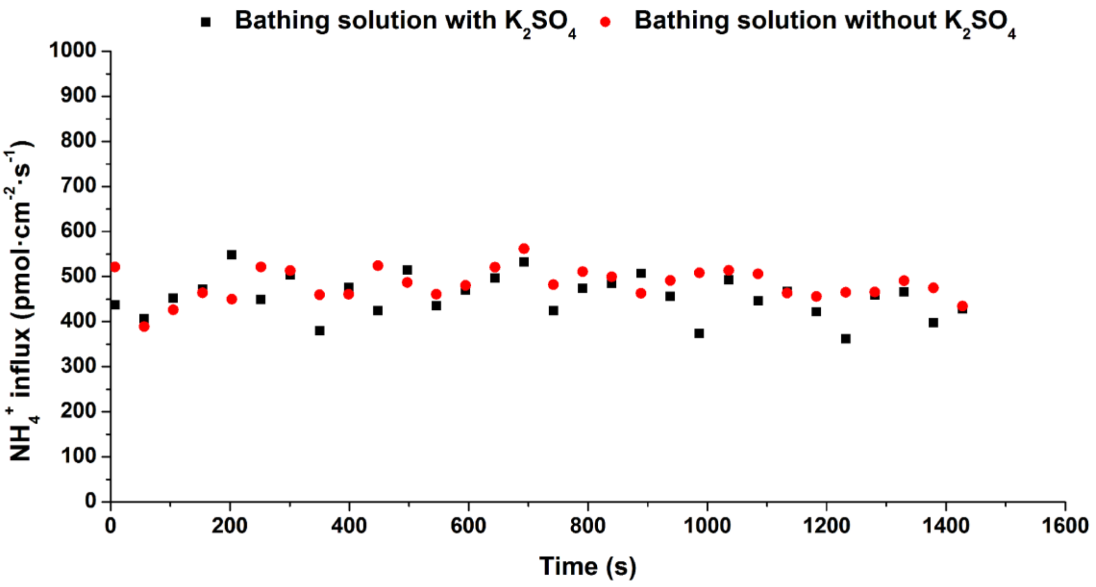

Supplementary Figure S4: The coefficient of variation under different balance time.

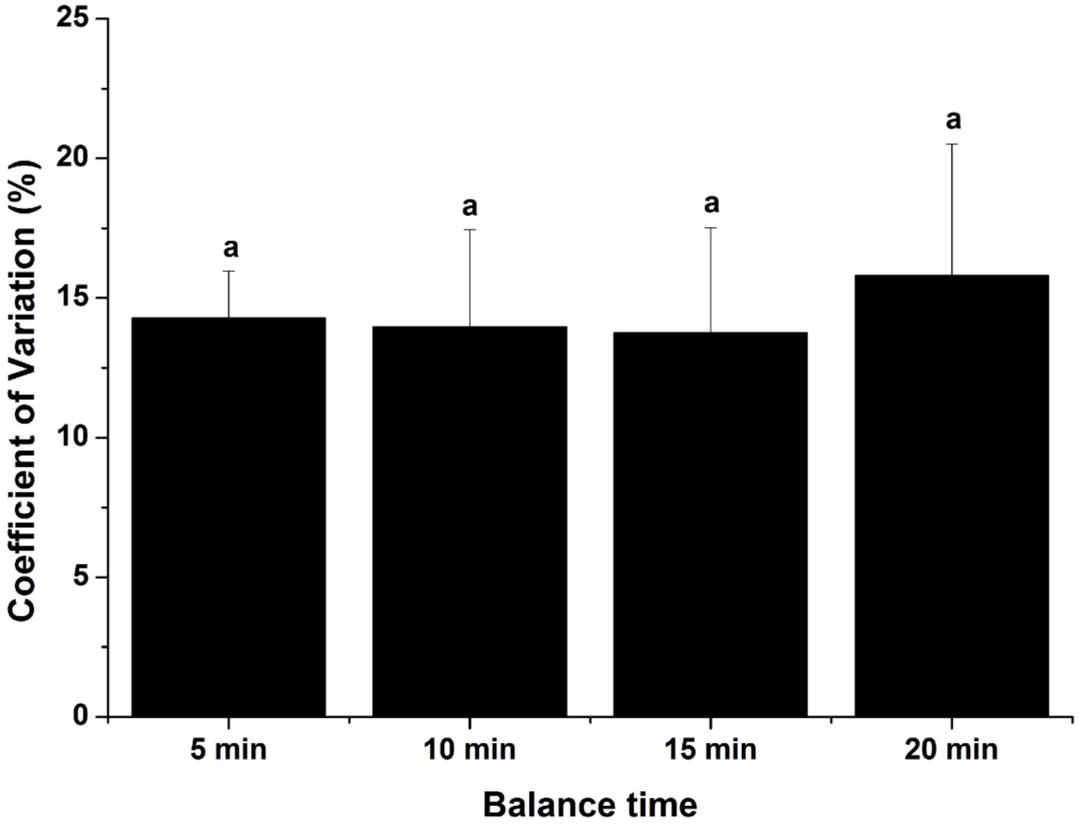

Supplementary Figure S5: Net NH_4^+ (a) andNO_3^- (b) fluxes along root tips of tea.

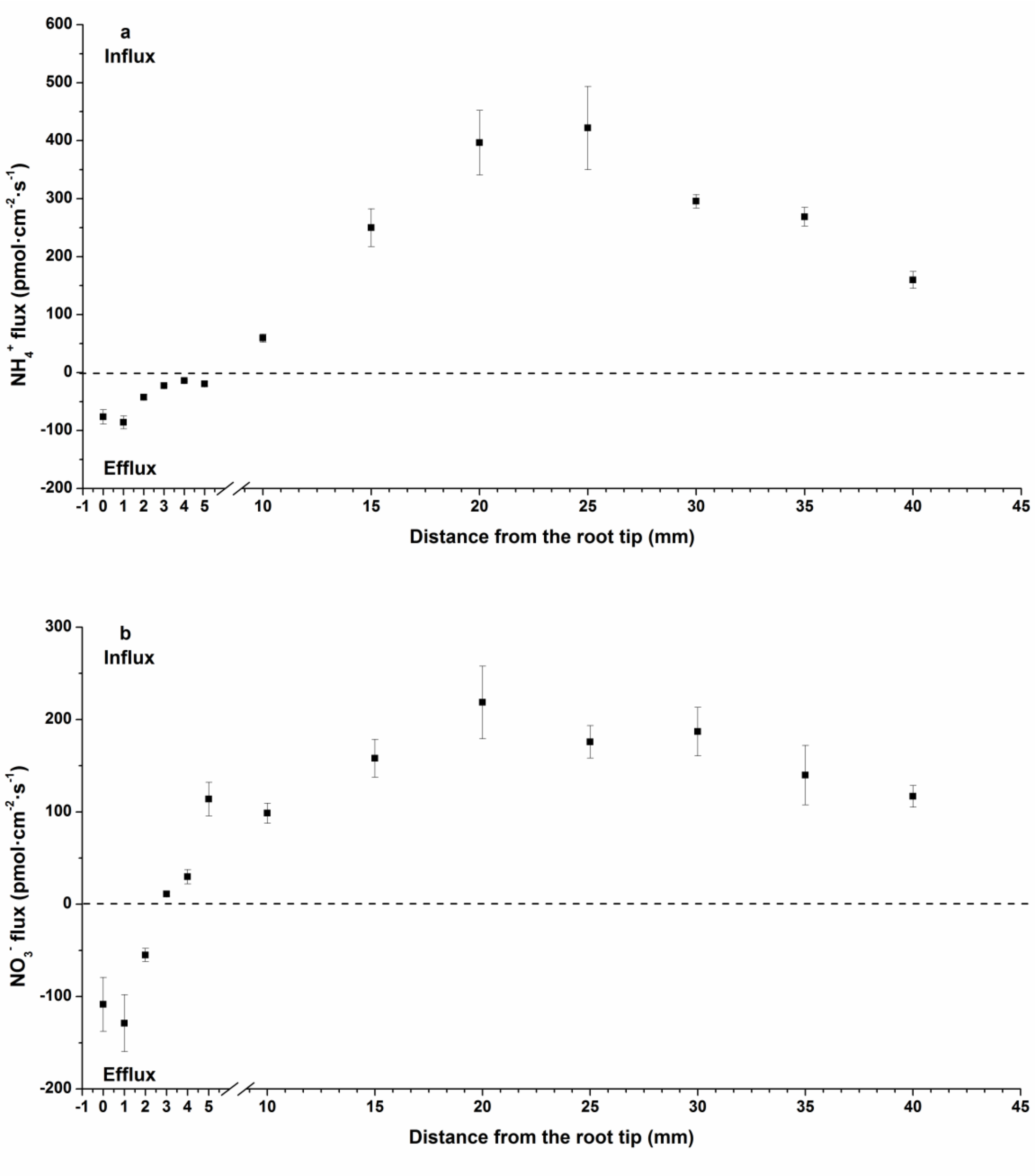

Supplementary Figure S6: Original recordings of all 6 biological replicates for NH_4^+ influx under single N form treatment.

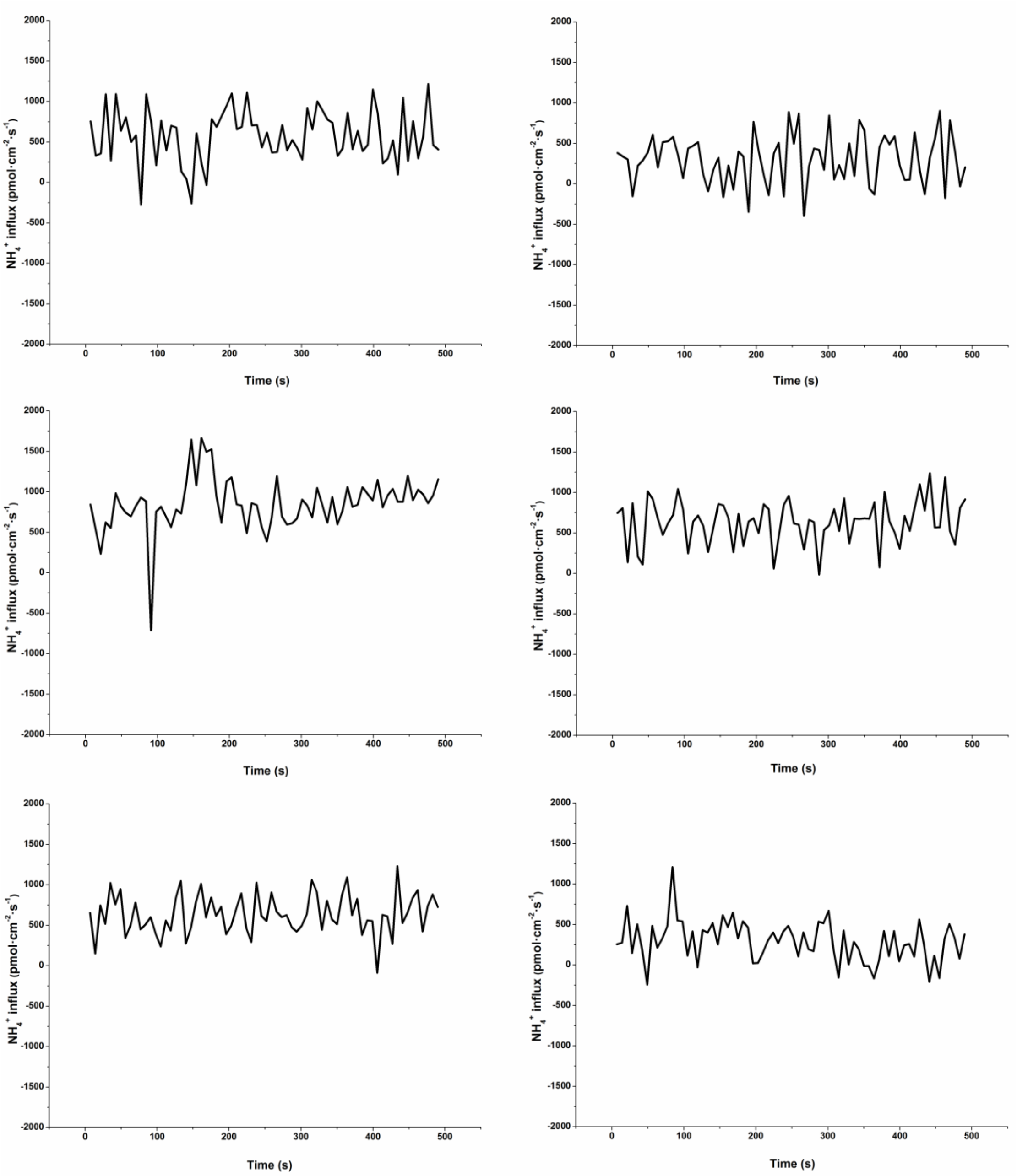

Supplement: Supplementary Information [file srep38370-s1.pdf]
